# Remarkable Symptoms Consequent upon an Injury Done to the Spine, with Remarks

**Published:** 1808-08

**Authors:** Jonathan Dorr

**Affiliations:** Cambridge, New York


Remarkable Symptoms *consequent upon an* Injury *done to the* Spine, *with Remarks. By* Jonathan Dorr, *Esq. of Cambridge, New York.*

On the 22d of July, 1794, A— A—, aged 36 years, received a blow across the small of his back by the fall of a tree, which partially dislocated his spine between the first and and second lumbar vertebræ, and which produced a considerable tumour, and an obtuse angle of the spine, with an entire abolition of all sensation and muscular action in the lower limbs, beginning on a circular line passing around the ossa innominata exactly over the head of the femur, and two inches above the ossa pubis. This was the lowest line of the least sensation, which was determined by puncturing the parts. As low down as another line, three inches above, sensation and muscular actiou was entire. From the upper to the lower line there was a gradual diminution of sensation. In this situation I visited him five hours after the accident. By adopting the method Mr. Bell directs, the bones were replaced. Stimulants were directed and applied. I visited him the succeeding day, and found his bladder violently distended, and a retention of fæces, without any more pain than a slight uneasiness about the region of the bladder. After drawing off his urine with a catheter, he became calm. This method of discharging his urine was followed about eight days, after which an involuntary discharge took place; but the rectum has never felt the irritation of distention since, as a manual operation has always been necessary to remove the fæces.

For about three years past his urine has been discharged in the following ways, viz. about one-fourth passed through a sinus, nearly in a lateral direction from the umbilicus, on the right side; one-third through the urethra; and the remainder through the most depending part of the scrotum, through several openings.

I called to see him, as usual, on the 4th of last Sept. at which time there was not the least vestige of a testicle to be felt. This circumstances led me to propose the following question, viz. whether he ever had any sexual inclination for his wife? He declared that idea was as obnoxious to his feeling as if she was of his own sex. I then inquired whether he ever saluted his wife with his lips? He told me that sensation was not entirely obliterated, but nearly so. The first was extinguished suddenly, and the latter progressively; and all the relative duties on his part were performed from a sense of the moral obligation he was under to her, and not from that peculiar affection that formerly made those duties pleasurable.

Mr. A's lower limbs are emaciated to a mere skeleton; his thighs always lying on a line with his body, and his legs longitudinally with his thighs, with his feet so drawn down as to be quite convex on their tops. Notwithstanding this this derangement and morbid condition of his lower limbs, above the upper line described before he is full fleshed, and every way firm and healthy as formerly, with a good appetite, and his mental powers as strong and active as ever, and sufficient strength in his arms to move himself out and into bed. To view him, one would imagine the two lines described above are two lines of demarkation, across which no influence could pass.

Does not the gradual abolition of excitement in the lips, following the entire destruction of excitement in the genitals, show the strong connection between those two organs, and help to illustrate the retrocession of the mumps from the throat to the scrotum, when too much exposed to cold, or when repellents are applied? If my patient is correct, how thrice happy are those who have a well-balanced system, that the duties of their lives, through the ills of time, may be made easy! What a weighty argument is this, to add to many others, to urge to strict economy for the preservation of health.

The retrocession of the mumps, or what Mr. John Hunter calls the "remote sympathetic operation," may proceed from two causes: the first from actual direct organical connection, which organical connection may serve as a medium of communication when a disease is repelled or recedes from one part of our system to another: secondly, from parts reciprocally and mutually irritated and excited into correspondent actions. All parts of our system are drawn into mutual consent by the stimulus of food. If this food produces a healthy action in the stomach, the same kind of action will be propagated through the system. From the perpetual stimulus of food, a perpetual correspondence and uniformity of action is kept up until a strong habit is established. This habit, joined to the sympathetic laws of the animal economy, will answer many singular purposes. I will here give one remarkable instance.—Six years ago I was stung by about thirty bees on my face and neck. I suddenly plunged myself into the water (which was at hand) to extricate myself from a general assault of the swarm. I remained in the water about half a minute. I then emerged from it, and instantly grew faint; and, all on a sudden, my whole surface, even to my legs and feet, exhibited all the characters and phenomena which the parts did that were actually stung, even to the puncture of the sting. This circumstance established me in the opinion, that inoculation with variolous matter, the bite of a rabid rabid dog, scarlet fever, &c. &c. were all local diseases, and that the system never became contaminated by any of these deleterious matters entering the mass of fluids, and there fermenting and assimilating them to its nature, but by propogating the same kind of action through the system that was begun in the point of insertion. The incipient inflammation will extend on the two principles laid down above, viz. by sympathetic laws, and the habit induced by stimulants.

If the system becomes contaminated by assimilation, I judge the work must commence immediately, and the matter suddenly be transferred to the system. To try the ground of these positions more amply by experiment, I vaccinated two persons; and thirty-six hours after vaccination, when a very slight flush appeared around the margin of the incision but no soreness under the arm, nor any other symptomof absorption, I applied a small bag of wet ashes, which destroyed the spot of puncture. In a few days a piece of dead flesh sloughed out one third of an inch in diameter, and half as deep as broad. Neither of these persons had the cow-pock.

If Dr. Mitchell's doctrine is true, that acids are the base of all poisons, I think there are strong reasons why alkaline escharotics should be preferred, and in a manner that should *rapidly* destroy to a sufficient extent. For it is certainly probable, that *gently* destroying the point of disease may increase excitability, and thereby call into action the inserted virus. The result of my experiment has made me extremely solicitous that some gentleman who may have an opportunity should try the method proposed in one of the volumes of the Medical Repository, of destroying the part bitten by canine rabies. The result of my experiments on the vaccinated persons has greatly strengthened my faith that the plan laid down could not fail of success.

*May* 23, 1803.

